# Understanding of the risk of HIV infection among the elderly in Ga-Rankuwa, South Africa

**DOI:** 10.1080/17290376.2014.931816

**Published:** 2014-06-24

**Authors:** Eucebious Lekalakala-Mokgele

**Affiliations:** ^a^ PhD, is Director of School of Health Care Sciences, University of Limpopo (Medunsa Campus), Pretoria, South Africa

**Keywords:** HIV infection, AIDS, risk, elderly, fears, HIV testing, infection par le VIH, le SIDA, le risque, les personnes âgées, la peur, le dépistage du VIH

## Abstract

The literature pertaining to the elderly shows that HIV infection among this population is on the increase, suggesting that the elderly population engages in activities risky for HIV infection. Reports on such behaviour include frequent sexual relations with much younger people and having multiple partners. A study was carried out in Ga-Rankuwa, a black township in Gauteng Province, South Africa to explore and describe the understanding of these elderly people regarding their risks of HIV infection and AIDS. Using a qualitative, exploratory design, three focus-group interviews were conducted with 32 women aged over 50 years. Findings revealed that older persons have knowledge about transmission of HIV infection and AIDS. However, a few had misconceptions as to how HIV infection is transmitted, as they believed that poor nutrition and sharing facilities play a role. Knowledge of mechanisms of protecting themselves against infection, such as use of a condom during coitus and wearing gloves when caring for infected family members, was also evident. The elderly indicated that they would prefer an older person, who they could identify with, to educate them more about HIV infection and AIDS. Although majority of participants had knowledge of how HIV is transmitted, and issues that put them at risk of transmission, a few the older persons had misconceptions about how HIV is transmitted due to lack of knowledge, as they believed that poor nutrition and sharing facilities can transmit infection. The lack of knowledge underscores the importance of addressing sexual risk with older people. It was very clear that more needs to be done in terms of education campaigns to dispel the myths of HIV infection and to empower the elderly.

## Résumé

La littérature relative aux personne âgées indique que l'infection à VIH est à la hausse parmi cette population, c'est qui suggère que la population âgée s'engage dans des activités a risque vis-à-vis de l'infection à VIH. Les publications ont mentionnées de genre de comportements incluant de fréquente relation sexuelles avec de jeunes gens et aussi ayant de multiple sexuel partenaires. Une étude avais été conduite en Garankuwa, un bas-lieu noir dans la province de Gauteng, en Afrique du Sud pour explorer et décrire la connaissance de ces personnes âgées concernant les risques qu'ils courent a l’égards de l'infection à VIH et du SIDA. En utilisant la perceptive qualitative de l'approche exploratoire, trois groupes d'entrevues ont été menées avec 32 femmes âgées de plus de 50 ans. Les résultants ont révélé que les personnes âgées ont suffisamment la connaissance sur la mode de transmission de l'infection à VIH et le SIDA. Cependant, quelques-uns parmi eux avaient de fausse compréhension sur la façon dont l'infection à VIH est transmise, car ils croient que la pauvre qualité alimentaire et le partage des installations sanitaires jouent un rôle. La connaissance de mécanismes pour se protéger contre l'infection, par exemple l'utilisation de condom préservatif lors du coït et de porter des gants pendant qu’ ils dispensent des soins aux membres de leur familles infectés était également évidente. Les personnes âgées ont indiqué qu'ils préféreraient une personne âgée et mature, à qui ils pourraient s'identifier, de leur éduquer davantage sur l'infection à VIH et le sida. Par conséquent, it était clair que le besoin en termes d’éducation de masse sur les mythes à propos de l'infection à VIH et le SIDA devrait être envisagé pour ce groupe à risque.

## Introduction

A growing proportion of HIV-infected individuals worldwide are now over the age of 50 years (Bhavan, Kampalath & Overton 2008). This increase in HIV-infected older people has particularly been observed in recent years in developed countries where the use of antiretroviral therapy is widespread (Bhavan *et al*. 2008; Navarro, Nogueras, Segura, Casabona, Miro & Murillas 2008; Orchi, Balzano, Scognamigli, Navarra, De Carli, Elia, *et al.* 2008; Pitts, Grierson & Misson 2005).

The South African national data on HIV prevalence for the 50 years and above age group show the increase to be 6.7% for the 50–60+-year-olds (Shisana, Rehle, Simbayi, Zuma, Jooste, Pillay-van-Wyk, *et al*. 2009). In South Africa, HIV prevalence differs markedly among the provinces, with the highest rates of new annual infections being in KwaZulu-Natal (16.7%) and Gauteng (16.0%) (Statistics South Africa 2013; Tomlinson 2006). Although statistics may indicate a slower HIV prevalence increase, older people are at risk of contracting HIV infection not only because they care for infected children, but also because of their own sexual behaviour (Lombard & Kruger 2009). The increase of AIDS in the elderly population suggests that they are engaging in activities that put them at risk for HIV infection (Cintrón-Bou 2004). Reports on such behaviour include frequent sexual relations with much younger people and a reluctance to use condoms (Andoulo, Meku & Léon 2004; Clüver, Elkonin & Young 2013; Muula 2008; Nkosana & Rosenthal 2007; Potgieter, Strebel, Shefer & Wagner 2012), being less concerned about being infected (Nguyen & Holodniy 2008), ignorance (Msobi, Fimbo & Msumi 2004; Negin, Nemser, Cumming, Lelerai, Amor & Pronyk 2012), and having multiple partners (Power, Bell & Freemantle 2010; Ramos Rodriguez, Baney, Morales, Parham & Lago 2000). The increase in HIV infection among this population group suggests the need to inquire whether they know about the risks of HIV infection and whether they perceive themselves to be at risk of infection.

Older persons tend not to think of HIV/AIDS as a problem relevant to their age group and are thus less likely to use condoms because they no longer worry about pregnancy (Nguyen & Holodniy 2008; Otani 2004). Frequency of sexual encounters into later periods of life is enhanced by the growth in use of impotence treatments, such as Viagra^©^ among elderly men, which unfortunately is dispensed without being accompanied by necessary messages about HIV infection prevention (Kanemoto 2003; Pantalone, Bimbi & Parsons 2008; Spearman & Bolden 2005; Spindler, Scheer, Chen, Klausner, Katz, Valleroy, *et al*. 2007). According to Schick, Herbenick, Reece, Sanders, Dodge, Middlestadt, *et al*. (2010) condom-use rates remain low across older age groups.

The literature indicates that the risk of infection and spread of HIV among older age groups goes undetected and unreported (AIDSInfoNet 2009; Fouad 2004). Increasing attention has been given to the indirect impacts of HIV infection, such as the effects of the epidemic on elderly people who are parents to those who are HIV positive and caregivers to affected grandchildren. However, little research has been conducted to understand how older persons consider themselves to be exposed to the risk of HIV infection. Such studies would be significant in shaping appropriate prevention interventions for older persons (Centers for Disease Control and Prevention 2008; Health Resources and Service Administration, HIV/AIDS Bureau 2009).

In her study Savasta (2004) suggested that there is inadequate HIV transmission education, poor awareness/risk perception, and insufficient patient/provider communication, all of which may contribute to the increased prevalence of HIV transmission in late middle-aged and older adults. Older people who lead an active sex life beyond their fertile years may not be concerned that their behaviour is risky (Skevington 2012). Although the incidence and prevalence of HIV transmission among the older population is startling, very little research has addressed knowledge about the risk and transmission of HIV in the African older population.

Research across generations has revealed differences between older adults and their younger counterparts in terms of sexual knowledge of risk behaviours, signifying the importance of age-appropriate interventions that target the specific needs of this unique cohort (Orel, Spence & Steele 2005). Research studies explaining the unique risk factors associated with transmission in this population are also limited (Savasta 2004). Hoffman (2003) alludes to the grand social narrative that older people are perceived to be sexually inactive, with the assumption that they are hence not HIV-infected or at risk of HIV infection. Although HIV secondary prevention efforts have been targeted at multiple at-risk populations, few intervention trials have focused specifically on the unique needs of older adults (Coleman, Jemmott, Jemmott, Strumpf & Ratcliffe 2009; Coon, Lipman & Ory 2003).

Results of the study conducted by Golub, Tomassilli, Pantalone, Brennan, Karpiak and Parsons (2010) underscore the importance of incorporating prevention information and interventions into standard medical care or social services programmes for older adults living with HIV. Fieldman (1994) insisted that most educational campaigns never show older adults, making them an ‘invisible’ at-risk population. As a result, older persons are generally less knowledgeable about HIV/AIDS than younger people and less aware of how to protect themselves against infection (Center for AIDS Prevention Studies 1997). Older people are also excluded from multimedia prevention campaigns, by virtue of the fact that the majority of older people may not be able to comprehend some of the HIV/AIDS prevention messages which usually target young people and high-risk groups (Chima, Chima & Azie 2004; Ingstad, Bruuns & Tlou 1997; Kirk & Goetz 2009; Williams & Tumwekwase 2001).

There is little research, however, to understand how older people consider themselves exposed to the risk of HIV infection. Such studies would be significant in shaping appropriate prevention interventions for older people (Chepngeno-Langat 2011). It is not known whether the elderly of Ga-Rankuwa community, a township in Gauteng province, have an understanding of the risk of HIV infection or not. Against this background, the following research question arises: What is the understanding of HIV risk among older persons in Ga-Rankuwa community?

The purpose of this study was to explore and describe the understanding of older persons regarding their risks of HIV infection and AIDS. The objectives were to: explore the understanding of HIV infection risk among older persons of Ga-Rankuwa community and describe the understanding of HIV infection risk among older persons of Ga-Rankuwa community.

## Methodology

### Study design

The study used a qualitative, explorative design involving persons aged 50 years and above attending luncheon clubs for older persons in Ga-Rankuwa, Gauteng Province, South Africa. The purpose of using an exploratory study was to investigate a little understood phenomenon (older persons' understanding of risk of HIV infection) to be able to identify important categories of meaning and to generate hypotheses for further research. The descriptive nature of the research was to describe and document how participants understood activities that could put them at risk of infection.

### Population and sampling

The population of this study was purposively selected to include older persons in the community, making use of luncheon clubs for the elderly in Ga-Rankuwa, as they would best contribute the information needed for the study (De Vos, Strydom, Fouché & Delport 2011:392; Polit & Beck 2006:271). A purposively selected group of 32 older persons from three luncheon clubs in Ga-Rankuwa participated in the study. The sample was selected on the basis of the following criteria: age of 50 years or more and attending luncheon clubs for the elderly in Ga-Rankuwa.

### Demographic data

Of the 32 participants interviewed, most (53.1%) were between the ages of 50 and 60 years.

Participants were all females, as no males were available to participate in the study. Participants were from different ethnic backgrounds: most (87.5%) were Setswana-speaking and were married (57.1%); 13 (40.6%) had primary education and 4 (12.5%) had no formal education (Table 1).

**Table 1.** Demographic data of the 32 older persons interviewed.

	No.	%
*Age (years)*		
50–60	3	9.4
61–70	17	53.1
71–80	11	34.3
81–90	1	3.1
*Ethnic language*		
Setswana	28	87.5
Sesotho	2	6.3
Sizulu	1	3.1
*Sex*		
All females	32	100.0
*Marital status*		
Single	7	21.8
Married	17	53.1
Widowed	3	9.4
Cohabiting	5	15.6
*Level of education*		
No formal education	4	12.5
Primary education	13	40.6
Secondary education	2	6.3
High school	12	37.5
Tertiary	1	3.1

### Bias

Sampling bias was avoided by carefully selecting participants based on their age and utilisation of luncheon clubs, and who thus represented the group of interest to the research. Interviews were held at a time and place convenient for participants and in a language of their own choice. Biased questioning was avoided by redirecting questions to other participants during probing, and the use of an independent coder assisted in prevention of interpretation bias.

### Setting

Focus-group interviews were conducted in the luncheon club (also known as a service centre) for older persons in Ga-Rankuwa. A luncheon club is a place – usually a church building, a clubhouse, a recreation hall or a home – where elderly people aged 50 and above meet once or twice a week. The purpose of the luncheon club is to cater for the social (companionship), emotional, cultural, physical, spiritual and educational needs of the aged. These clubs are run by old people themselves in partnership with community groups and a social worker.

### Data collection

Three focus-group discussions were conducted, with two groups consisting of 10 participants each and 1 of 12 participants. The groups were large enough to provide a diversity of understanding of the phenomenon and small enough to enable all participants the opportunity to share their insights. Data were collected on days that older persons were participating in their scheduled activities. The managers of the luncheon clubs assisted the researcher in organising the venue and inviting participants to take part. The dates and times were arranged with the managers of the luncheon clubs and the older persons so that daily activities were not interrupted. The research assistant explained the purpose of the research and the process of data collection.

Interviews were conducted in Setswana, which is the language predominately spoken in Ga-Rankunwa community. Even though participants were from different ethnic backgrounds, they all spoke Setswana very well and agreed among themselves that they preferred the interviews to be conducted in Setswana. Data were tape-recorded and transcribed verbatim. Field notes were written by the researcher while the research assistant was conducting the interviews, to augment these data and to record non-verbal cues and details.

An interview guide was used by a research assistant to collect the data. The interview guide contained questions about participants' understanding of HIV and the risk of infection with HIV. Participants were first asked to explain how, in their understanding, a person can be infected with HIV, as the leading question to obtain their background knowledge on HIV infection. They were then asked the following questions:

- Please explain to me what you know or understand to be the risk of HIV infection among your age group?
- How can older persons protect themselves from being infected with HIV?
- What are your opinions about testing for HIV infection?
- What can be done to increase your knowledge on HIV risk factors?

The researcher probed using different strategies, such as repeating the question, using silent probes and repeating the answers, which provided confirmation of responses.

### Data analysis

Before data analysis, a research associate fluent and competent in both Setswana and English translated all Setswana interview transcripts verbatim into English. Thereafter, the data were manually analysed by the researcher using a combination of the methods of Tesch (1990 cited in Creswell 2009:185) and Giorgi (1970 as quoted by Omery 1983). The researcher read through all of the transcripts to get a sense of the whole, and thoughts were written in the margins. Similar concepts were clustered together. The data were then read through for the second time, comparing them to the list of concepts identified to form categories. Data belonging to the same category were assembled. Redundant information was identified and eliminated. Data were integrated and synthesised into a descriptive structure and codes were created.

### Trustworthiness

The principles of trustworthiness as described by Lincoln and Guba (1985) were applied. Credibility was ensured by member checking of descriptions of participants of their understanding of risks of infection. Member checking was done throughout the interviews by probing and repeating questions to other members of the focus group and allowing participants to corroborate information. Participants could approve or disapprove particular aspects of the interpretation of the data provided (Doyle 2007). Prolonged engagement with the data allowed the researcher to identify recurring patterns and themes. Use of an independent coder skilled in the field of research assisted in ensuring dependability. Tape-recorded data and field notes were kept as an audit trail to ensure confirmability.

### Ethical considerations

The nature and the scope of the study were explained to the participants (in the language of their choice) who gave their informed consent. Participants were informed that participation was voluntary and that they had the right to discontinue their participation if they felt uncomfortable with the topic under discussion or did not wish to continue. Informing participants about the study purpose and their right to withdraw ensured that their rights were respected. Interviewing participants from different ethnic backgrounds ensured fairness in selection. Approval for the study was granted by the managers of luncheon clubs and the elderly persons as well as the Ethics Committee of the University of Limpopo (Medunsa campus) before data collection.

## Findings

Four themes emerged from the interviews: knowledge of how HIV infection occurs; protection against infection; need for testing; and desire for age-appropriate prevention strategies. Table 2 illustrates the categories within the themes.

**Table 2.** Themes and categories of analysed data.

Themes	Categories
Knowledge of HIV infection	Knowledge of mechanism of infection
	Misconception of infection
	Dispute of misconceptions
Protection against HIV infection	Protection methods
	Challenges on condom usage
Testing for HIV infection	The need to test
	Knowledge of the window period
	Fears related to testing
Age-appropriate strategies	Information appropriate for the aged

### Knowledge of HIV infection

Findings showed that most of the participants had an understanding of how people become infected with HIV. Under this theme three sub-themes emerged: knowledge of the mechanism of infection; misconceptions of infection; and dispute of misconceptions.

#### Knowledge of mechanism of infection

A number of practices that could put older persons at risk of HIV infection were revealed during the focus-group interviews. These included unprotected sex, caring for infected family members without protective wear, and using the same needles or sharp objects. Quotes supporting this theme include the following:

By having sex without protection … that's how you get it … flesh to flesh …  (P1, Group 1)

I think when you're dealing with an infected person … and you handle them with your bare hands … you expose yourself when you wash them because they have sores and cuts … only to find that you get infected … then it seems as if you as an elderly woman you were unfaithful …  (P3, Group 2)

 … if you make the mistake of not changing the needle and use on me with HIV … and then go and use it again on someone else … they may be infected … thereby spreading the virus. (P2, Group 3)

#### Misconceptions of infection

Even though the majority understood how individuals can become infected, there were a few participants whose knowledge about how HIV infection occurs was not correct. Some misconceptions mentioned by participants were the role of poor diet, and believing that HIV can be transmitted by air and by sharing facilities. The following quotes attest to the above:

I still maintain that we don't eat nutritious food … so you find that our blood … the red blood cells … and white blood cells … you find that one is weak between them … because there is nothing stopping the virus if you are not eating well. You don't eat fruits … only starch every day … another thing … we eat chicken feet too much… and the intestines too much … rather buy instead, you enjoy a nice meal …  (P1, Group 2)

Also food … the food we eat lacks vitamins … and we blame poverty, whereas that's not the cause … we eat too much starch … we don't eat our spinach … boiled vegetables so we don't fall ill … we eat junk food. (P17, Group 2)

Also if you go into the toilet directly after an infected person had used it, you will get infected. (P24, Group 3)

 … their smell … being close to them … you breathe their breath and so on …  (P12, Group 2)

#### Dispute of misconceptions

Some participants disputed these misconceptions and corrected those that believed these myths, as evident in the following:

The food she's referring to, that we don't eat nutritiously, it's when you already have HIV that you should eat nutritiously so that your immunity is strong. It matters when you are infected, but if you are not infected and you eat recklessly … there is no HIV in food. (P9, Group 1)

I think our poverty as black people is the reason we don't eat nutritiously … that's what kills our bodies. We hear all the time that at least eat a fruit a day, to build your immunity. (P8, Group 1)

I want to raise the toilet issue, that if an infected person had just used it, and you follow, that you get infected. I don't agree with that one … because if that was the case, then they would have to be separated (quarantined) from the uninfected. (P26, Group 3)

### Knowledge on protection against HIV infection

Two sub-themes emerged from this theme, namely ways of protecting themselves and challenges with the use of condoms.

#### Ways of protecting against infection

Participants mentioned multiple ways of protecting themselves against infection. These included the use of condoms, abstinence, wearing gloves when caring for infected people, and avoiding kissing. The following excerpts illustrate this:

When it comes to sex we must use condoms, you cannot just expose yourself to your partner because you don't know what disease they have. Otherwise I prefer to abstain, when he comes home late. You will suspect something … you start sleeping in your tights, so he can't touch you. (P7, Group 1)

Also through handling an infected person, you must know what to use. You must be sure to wear your gloves before you handle them. (P15, Group 2)

I no longer prefer to kiss, because it may happen that someone's lips are cracked and they know that they are infected and they kiss you intentionally. Even on the cheek, I don't want to be kissed. (P23, Group 3)

#### Challenges of condom usage

Participants cited that although they knew they needed to protect themselves, some partners refused to use condoms. One feared that the condom will burst, while another was embarrassed to be found out if family members realised that they are sexually active and using condoms. It seemed that these female participants encouraged each other not to agree to using condoms, as they apportioned blame to the unfaithful partners:

These old men don't want to use condoms … he'll tell you that ever since they were born, they've never had to use the stuff. (P5, Group 1)

Next thing, the condom bursts and makes matters worse … we as women must not agree …  (P6, Group 1)

I wouldn't agree to the condom … he was the unfaithful one, not me. Even my daughters-in-law will see these things in my bedroom [when] cleaning it … (P8, Group 1)

### Testing for HIV infection

Participants were aware of the need to test and the reasons for doing so. Three sub-themes emerged from this theme, namely the need for testing, knowledge of the window period, and the fear of testing.

#### The need to test

Older persons in this study understood the need to be regularly tested for HIV infection. Participants were of opinion that they needed to know their own status and acknowledged that they too can be infected. They also indicated that nurses advised them to get tested:

Before you meet there you have to go and get tested, because the danger is in the blood. (P22, Group 3)

The boyfriend must get tested, me too, because we like ‘flesh to flesh’ … before we go flesh to flesh …  (P9, Group 1)

The nurses would ask me to also test …  (P15, Group 2)

Two participants who were traditional healers indicated that they have to test more frequently as a result of the nature of their work, as they often have to wash the soiled clothes of their patients without any protection:

I work with people, I cannot just leave them. Sometimes they have soiled themselves up. (P9, Group 1)

I have to take off the clothes and wash them, and then dry them when they have soiled themselves. That's why I test every second month … I don't have gloves. (P20, Group 2)

We deal with all sort of ages, sometimes a baby that looks healthy is brought to you and you handle it and give her an enema without knowing she is already sick, so I have to test frequently. (P32, Group 3)

#### Knowledge of window period

Some participants even understood the issue of the window period and its possible impact:

Even after you have tested, it doesn't end there because you could find that you are negative … but you could get infected after a few weeks, so you must go back again to get tested. (P30, Group 3)

We are supposed to get tested first … after testing, we should observe the waiting period they told us about, so we see what happens. (P32, Group 3)

#### Fears related to testing

Even though older persons understood the issue of testing, the fear of testing was also brought up during interviews:

I've realised that we elderly … are afraid to get tested, but that's where you could get helped. I went, because when you have it, you have it …  (P14, Group 2)

But it's not as simple as that … It's not easy to get tested in the first place, it can't simply be easy after you tested. It is scary to test, as we say that we may not know our statuses, so it is scary if you consider the question: ‘What if you are infected?’ What will you do, who will you tell? You will have such mixed feelings … because you don't know, and some don't have knowledge, they have never been told about such aspects … it can be disastrous. (P5, Group 1)

Then after being told, it gets worse than before you went, you start thinking your life is over until they come and counsel you, because we associate HIV with death. (P26, Group 3)

### Age-appropriate prevention strategies

Participants indicated that health providers in the primary healthcare facilities informed them about HIV/AIDS, its prevention and how to protect themselves. They all were concerned by the lack of prevention strategies that are appropriate to their age group (unlike what is the case for younger people). They also indicated the desire to have an aged person teaching them about HIV prevention, and that the teaching should take place in the community, luncheon clubs, at pension points and in churches:

I prefer if it's done by older people … I don't look down on younger people, they know more, but I prefer being taught by older people. (P9, Group 1)

We can also go and spread the word among other elderly how about doing this more regularly maybe that will help. The information must be shared with more people than us sitting here. If the nurse at the clinic did not tell me about AIDS I would have not known that it affects people as old as I am. Television only shows young people. (P12, Group 2)

I'd like to add on to what she is saying. We're in social clubs, we attend funerals. At memorial services, first thing, it is mentioned to elderly women [that] we can all see that people are dying. Let's go to clinics and test so we know our statuses, talk about diseases like TB, arthritis; include all the diseases, because if you single out one disease like HIV, they won't come. If you tell them that they will be addressed by experts on all these diseases, they will come to hear for themselves, and their eyes will be opened and they will get tested. (P24, Group 3)

## Discussion

This study set out to describe the understanding of the risk of HIV infection among older persons in the Ga-Rankuwa community. The results of the data analysis show that older persons have knowledge of the transmission of HIV from person to person. About a decade ago Akwara, Madise and Hinde (2003) were of the opinion that while knowledge of AIDS has increased remarkably over the years, and is almost universal in most sub-Saharan African countries, the association between such knowledge and sexual behaviour was still rather ambiguous.

Most participants had the correct information on how person to person transmission of HIV infection occurs, although some believed that sharing utensils can promote transmission of infection. Some participants disputed such myths, which may be an indication that there is some understanding among the elderly of the risks of infection. Myths or fallacies of HIV transmission have been documented elsewhere (Genrich, Brader & Brathwaite 2005), and continued belief in them seen in this study underscores the importance of addressing sexual risk among older people.

Participants also understood that caring for infected people without any protective clothing can transmit infection. However, the lack of resources while caring for their infected families could predispose them to becoming infected. This aspect needs to be considered for this group of adults, as the literature highlights that one of the indirect impacts of HIV is elderly caregivers (Lalthapersad-Pillay 2008; Rajaraman, Earleb & Heymann 2008; Schatz 2007). Chepngeno-Langat (2011) is of the opinion that older persons have been seen as carers of orphans and people living with AIDS and hardly as people at risk of HIV infection. It is important that older persons as carers and as human beings have the right to be protected and to be given resources to protect themselves during care of their sick family members.

The literature has advocated the use of condoms as a mechanism of protection against HIV infection (Bankole, Singh, Hussain & Oestreicher 2009; Feldblum, Welsh & Steiner 2003), and participants generally supported this notion. However, some had challenges with the use of condoms – for example, husbands refusing to use them. The challenges of condom usage and inability to negotiate condom use can contribute to older women being at risk of HIV infection. This phenomenon is not only common in the elderly but has also been found in the younger generation and is associated with gender inequalities that place women in a subordinate position. This contributes significantly to women's inability to negotiate the use of condoms, and may be the central obstacle to AIDS prevention in Africa (Akwara *et al*. 2003; Exavery, Kanté, Jackson, Noronha, Sikustahili, Kassimu Tani, *et al.* 2012). According to Exavery *et al.* (2012) and Shisana, Zungu-Dirwayi, Toefy, Simbayi, Malik and Zuma (2004), confidence to negotiate safer sex practices is crucial, especially today when AIDS due to HIV is rampant.

The issue of the embarrassment of being found out to be using condoms by other family members is related to ageist and sexist stereotypes which perpetuate the myth that the elderly are not sexually active (Shisana *et al.* 2004; Spearman & Bolden 2005). The fear that the condom might burst relates to doubting the efficacy of the condom as a method of preventing infection, which according to Versteeg and Murray (2008) is a barrier to the use of condoms. This can perpetuate HIV infection since without protection one is exposed.

Participants believed that abstinence and self-control were good ways of preventing infection, similar to findings in other African studies (Akwara *et al.* 2003; Chepngeno-Langat 2011; Shisana *et al.* 2004). In Ward, Disch, Levy and Schensul's (2004) study, some participants even expressed their intention of becoming celibate for life because of fear of contracting AIDS.

Testing for the presence of HIV was also understood as important to preventing the risk of infection; many indicated that they would do so if advised by nursing staff in the local clinic to have themselves tested. Studies attest that older persons are not likely to take an HIV test unless prompted by a healthcare person (Akers, Bernstein, Henderson, Doyle & Corbie-Smith 2007; Lekas, Schrimshaw & Siegel 2005). The persistence of high levels of stigma among the elderly women can reduce the possible uptake of HIV testing programmes and lead to reduced care-seeking behaviour (Negin *et al*. 2012). In the opinion of Deblonde, De Koker, Hamers, Fontaine, Luchters and Temmerman (2010) it appears that low risk perception constitutes a barrier to HIV testing among HIV-infected individuals. The second barrier is associated with fear and worries. While they had understanding of testing for the presence of HIV, participants in this study were also fearful of the results. Uncertainty about the perceived ability to cope with a positive result, leading to fear, is highlighted as an important barrier to HIV testing. In addition, the perception of HIV as a deadly rather than a chronic, manageable disease is an important cause of fear (Flowers, Duncan & Knussen 2003). AIDS in South Africa carries with it a stigma related to fear of dying and death (Macintyre, Rutenberg, Brown & Ali Karim 2003). Understanding the perceptions of older persons relating to testing and its barriers is critical to improving effectiveness of HIV testing and counselling for this particular group.

Risk perception has been theorised as an important antecedent for adopting protective behaviour (Macityre *et al.* 2003). Older persons in the present study wished to have more information regarding how they can protect themselves. They asserted that an older person that they could identify with would be ideal for educating them about issues related to HIV/AIDS. Ward *et al.* (2004) opine that education interventions designed for older adults must emphasise that it is behaviour that puts people at risk, and not *who they are or who they perceive themselves to be*. Furthermore, persisting misconceptions about transmission of HIV/AIDS should be identified and explicitly addressed, especially in older populations for whom language and other cultural barriers result in low access to prevention messages delivered through mainstream media communications.

## Conclusion

The findings of this study show that older persons' knowledge about HIV/AIDS transmission is high, including knowledge of the risks of unprotected sex and exposure to infected blood while caring for the injured and sick. A few of the older persons had misconceptions about how HIV is transmitted due to lack of knowledge, as they believed that poor nutrition and sharing facilities can transmit infection. The lack of knowledge underscores the importance of addressing sexual risk with older people. Participants also understood mechanisms of protecting themselves against infection, such as use of condom during coitus and wearing gloves when caring for infected family members (which is important in reducing infection during care).

Resistance by male partners to using condoms was mentioned, as well as the fear of condoms bursting. The issue of embarrassment if relatives found out that they were using condoms was highlighted, and this is associated with the myth that older persons are asexual. Participants understood the need to test and indicated that that nurses also advise them to go for testing. This aspect is important, as those who perceive themselves to be at low risk of infection may not subject themselves to testing and thus will not know their status and consequently will perpetuate new infections.

Some participants even understood the window period; this aspect needs more attention as older persons may assume that they are negative when they are actually infected. The fear of testing was mentioned and requires that older persons are well counselled and encouraged to test for early detection and treatment. Findings of this study have implications for HIV services for older persons. HIV responses need to account for older persons by reflecting risks and trends providing appropriate education, prevention and testing services for this population. It was very clear that more needs to be done in terms of education campaigns to dispel the myths of HIV infection and to empower the elderly.

## Recommendations

The following recommendations flow from the conclusions; that

1. Education campaigns be conducted by older persons who the elderly population can identify with. These campaigns should be carried out where the elderly meet on a daily basis.
2. Older adults should play a critical role as educators of other older people and should remain influential community members.
3. Age-appropriate advertising campaigns targeting older persons should form part of the education campaign. Measures that can assist in dispelling myths about HIV should be emphasised during campaigns.
4. Healthcare providers, including social workers, should begin to facilitate open discussions with the elderly about their health and sexual history on all platforms of engagement, be it at primary healthcare centres, hospitals or luncheon clubs. They should be aware of their own comfort level in talking about sexuality, HIV/AIDS and ageing.
5. A comprehensive biopsychosocial-spiritual assessment, which includes questions about sexual practices and alcohol and drug use, and creates an opportunity to talk with clients about HIV transmission risk, should be carried out when older persons consult healthcare providers.
6. Discussions with the elderly should also include sharing of harm reduction practices and strategies that address alcohol use and safer sex to promote HIV prevention.

## Limitations of the study

The findings of this study do not reflect the knowledge of all of the elderly population of Ga-Rankuwa and thus cannot be generalised. This study only reflects the views of older female participants, since no males made use of the luncheon clubs at the time the research was conducted. It would have been more enlightening to also know the views of older men in comparison to their female counterparts.
